# Supplementary material for: Zeta Inhibitory Peptide attenuates learning and memory by inducing NO-mediated downregulation of AMPA receptors
Source: Nat Commun. 2020 Jul 23;11:3688. doi: 10.1038/s41467-020-17484-y (PMC7378180; doi:10.1038/s41467-020-17484-y)
Supplement: Supplementary file 2 — Reporting Summary [file 41467_2020_17484_MOESM2_ESM.pdf]

nature research

Corresponding author(s): Rami Yaka

Last updated by author(s): Jun 24, 2020

Reporting Summary

Nature Research wishes to improve the reproducibility of the work that we publish. This form provides structure for consistency and transparency in reporting. For further information on Nature Research policies, see Authors & Referees and the Editorial Policy Checklist .

Statistics

For all statistical analyses, confirm that the following items are present in the figure legend, table legend, main text, or Methods section.

n/a	Confirmed	
☐	☒	The exact sample size (*n*) for each experimental group/condition, given as a discrete number and unit of measurement
☐	☒	A statement on whether measurements were taken from distinct samples or whether the same sample was measured repeatedly
☐	☒	The statistical test(s) used AND whether they are one- or two-sided<br>*Only common tests should be described solely by name; describe more complex techniques in the Methods section.*
☒	☐	A description of all covariates tested
☐	☒	A description of any assumptions or corrections, such as tests of normality and adjustment for multiple comparisons
☐	☒	A full description of the statistical parameters including central tendency (e.g. means) or other basic estimates (e.g. regression coefficient) AND variation (e.g. standard deviation) or associated estimates of uncertainty (e.g. confidence intervals)
☐	☒	For null hypothesis testing, the test statistic (e.g. *F*, *t*, *r*) with confidence intervals, effect sizes, degrees of freedom and *P* value noted<br>*Give P values as exact values whenever suitable.*
☒	☐	For Bayesian analysis, information on the choice of priors and Markov chain Monte Carlo settings
☒	☐	For hierarchical and complex designs, identification of the appropriate level for tests and full reporting of outcomes
☒	☐	Estimates of effect sizes (e.g. Cohen's *d*, Pearson's *r*), indicating how they were calculated

Our web collection on statistics for biologists contains articles on many of the points above.

Software and code

Policy information about availability of computer code

Data collection	p-Clamp v10 (electrophysiology), Olympus Fluoview FV10-ASW v4.2 (confocal images), BioRad ImageLab V5 (western blot)
Data analysis	Image J 1.53, Graphpad Prism v8, Biorad ImageLab V5

For manuscripts utilizing custom algorithms or software that are central to the research but not yet described in published literature, software must be made available to editors/reviewers. We strongly encourage code deposition in a community repository (e.g. GitHub). See the Nature Research guidelines for submitting code & software for further information.

Data

Policy information about availability of data

All manuscripts must include a data availability statement. This statement should provide the following information, where applicable:

- Accession codes, unique identifiers, or web links for publicly available datasets
- A list of figures that have associated raw data
- A description of any restrictions on data availability

All uncropped blots, electrophysiology and behavioral data are provided as a Source Data File. Further data are available on request from the authors.

Field-specific reporting

Please select the one below that is the best fit for your research. If you are not sure, read the appropriate sections before making your selection.

☒ Life sciences ☐ Behavioural & social sciences ☐ Ecological, evolutionary & environmental sciences

October 2018

For a reference copy of the document with all sections, see nature.com/documents/nr-reporting-summary-flat.pdf

Life sciences study design

All studies must disclose on these points even when the disclosure is negative.

Sample size	Sample sizes are based on previously published studies (Schumann et al., J Neurosci 2009)
Data exclusions	No data were excluded
Replication	Biological replicates were performed for all experiments and the number N shown for each in the Figure Legend
Randomization	Male rats were used throughout the study aged between 6 and 8 weeks and randomly assigned to saline, peptide and cocaine treatments. For primary neurons experiments, cells from each litter were pooled and randomly allocated into treatment groups.
Blinding	The in vivo experiment was performed in a semi-blind fashion, in which technicians injected the mice and the analysis was done by students who were not familiar with the outcome. Blinding for electrophysiological studies was not possible. However, in vitro confocal analysis was performed semi-blind as the analysis was carried out by a researcher who was not aware of the group allocation.

Reporting for specific materials, systems and methods

We require information from authors about some types of materials, experimental systems and methods used in many studies. Here, indicate whether each material, system or method listed is relevant to your study. If you are not sure if a list item applies to your research, read the appropriate section before selecting a response.

Materials & experimental systems

n/a	Involved in the study	
☐	☒	Antibodies
☐	☒	Eukaryotic cell lines
☒	☐	Palaeontology
☐	☒	Animals and other organisms
☒	☐	Human research participants
☒	☐	Clinical data

Methods

n/a	Involved in the study	
☒	☐	ChIP-seq
☒	☐	Flow cytometry
☒	☐	MRI-based neuroimaging

Antibodies

Antibodies used

anti-GluA1 (extracellular domain): GluA1-N355/1 – Abcam [ab174785], anti-GluA1: Mercury [AB1504], anti-GluA1-pS831: Mercury [04-823], anti-PKCZ: Abcam [ab59364], anti-PKCZ-pThr403/410: Cell Signalling [9378], anti-PKMZ: Mercury [07-264], anti-PKC-iota: Abcam [ab53878], anti-actin: Abcam [ab8227], anti-tubulin: Sigma [T9026]

Validation

Validation of all primary antibodies used in this study based on manufacturers' statements published on their websites:
anti-GluA1-N355/1 – Abcam [ab174785] https://www.abcam.com/glutamate-receptor-1-ampa-subtype-antibody-n3551-n-terminal-ab174785.html
anti-GluA1: Mercury [AB1504] https://www.merckmillipore.com/INTL/en/product/Anti-Glutamate-receptor-1-Antibody,MM_NF-AB1504
anti-GluA1-pS831: Mercury [04-823] https://www.merckmillipore.com/INTL/en/product/Anti-phospho-GluR1-Ser831-Antibody-clone-N453-rabbit-monoclonal,MM_NF-04-823
anti-PKCZ: Abcam [ab59364] https://www.abcam.com/pkc-zeta-antibody-ab59364.html
anti-PKCZ-pThr403/410: Cell Signalling [9378] https://www.cellsignal.com/products/primary-antibodies/phospho-pkcz-l-thr410-403-antibody/9378
anti-PKMZ: Mercury [07-264] https://www.mercury-ltd.co.il/antibodies_and_assays_07264_1
anti-PKC-iota: Abcam [ab53878] https://www.abcam.com/pkc-iota-antibody-ab53878.html
anti-Actin: Abcam [ab8227] https://www.abcam.com/beta-actin-antibody-ab8227.html
anti-tubulin: Sigma [T9026] https://www.sigmaaldrich.com/catalog/product/sigma/t9026

Eukaryotic cell lines

Policy information about cell lines

Cell line source(s)	HEK293 cells were sourced from ATCC catalog # CRL-1573
Authentication	Authentication was performed by analysis of morphology

Mycoplasma contamination	Mycoplasma contamination was not detected in this cell culture
Commonly misidentified lines (See ICLAC register)	*Name any commonly misidentified cell lines used in the study and provide a rationale for their use.*

Animals and other organisms

Policy information about studies involving animals; ARRIVE guidelines recommended for reporting animal research

Laboratory animals	Male Sprague Dawley rats (20-35 days) were used for electrophysiology and injection of peptides. C57Bl6 pregnant dams (E14) were used for mouse primary cell culture.
Wild animals	No wild animals were used in this study
Field-collected samples	No field-collected samples were used in this study
Ethics oversight	The Institutional Animal Care Committee (IACUC) of the Hebrew University (Jerusalem, Israel) approved all procedures.

Note that full information on the approval of the study protocol must also be provided in the manuscript.
